# Does a Deep Learning–Based Computer-Assisted Diagnosis System Outperform Conventional Double Reading by Radiologists in Distinguishing Benign and Malignant Lung Nodules?

**DOI:** 10.3389/fonc.2020.545862

**Published:** 2020-10-09

**Authors:** Zhou Liu, Li Li, Tianran Li, Douqiang Luo, Xiaoliang Wang, Dehong Luo

**Affiliations:** ^1^Department of Radiology, National Cancer Center, National Clinical Research Center for Cancer, Cancer Hospital & Shenzhen Hospital, Chinese Academy of Medical Sciences, Peking Union Medical College, Shenzhen, China; ^2^Department of Radiology, National Cancer Center, National Clinical Research Center for Cancer, Cancer Hospital, Chinese Academy of Medical Sciences, Peking Union Medical College, Beijing, China; ^3^Department of Pathology, National Cancer Center, National Clinical Research Center for Cancer, Cancer Hospital & Shenzhen Hospital, Chinese Academy of Medical Sciences, Peking Union Medical College, Shenzhen, China

**Keywords:** computer-assisted diagnosis, deep learning, solitary pulmonary nodules, malignancy, differential diagnosis

## Abstract

**Background:**

In differentiating indeterminate pulmonary nodules, multiple studies indicated the superiority of deep learning–based computer-assisted diagnosis system (DL-CADx) over conventional double reading by radiologists, which needs external validation. Therefore, our aim was to externally validate the performance of a commercial DL-CADx in differentiating benign and malignant lung nodules.

**Methods:**

In this retrospective study, 233 patients with 261 pathologically confirmed lung nodules were enrolled. Double reading was used to rate each nodule using a four-scale malignancy score system, including unlikely (0–25%), malignancy cannot be completely excluded (25–50%), highly likely (50–75%), and considered as malignant (75–100%), with any disagreement resolved through discussion. DL-CADx automatically rated each nodule with a malignancy likelihood ranging from 0 to 100%, which was then quadrichotomized accordingly. Intraclass correlation coefficient (ICC) was used to evaluate the agreement in malignancy risk rating between DL-CADx and double reading, with ICC value of <0.5, 0.5 to 0.75, 0.75 to 0.9, and >0.9 indicating poor, moderate, good, and perfect agreement, respectively. With malignancy likelihood >50% as cut-off value for malignancy and pathological results as gold standard, sensitivity, specificity, and accuracy were calculated for double reading and DL-CADx, separately.

**Results:**

Among the 261 nodules, 247 nodules were successfully detected by DL-CADx with detection rate of 94.7%. Regarding malignancy rating, DL-CADx was in moderate agreement with double reading (ICC = 0.555, 95% CI 0.424 to 0.655). DL-CADx misdiagnosed 40 true malignant nodules as benign nodules and 30 true benign nodules as malignant nodules with sensitivity, specificity, and accuracy of 79.2, 45.5, and 71.7%, respectively. In contrast, double reading achieved better performance with 16 true malignant nodules misdiagnosed as benign nodules and 26 true benign nodules as malignant nodules with sensitivity, specificity, and accuracy of 91.7, 52.7, and 83.0%, respectively.

**Conclusion:**

Compared with double reading, DL-CADx we used still shows inferior performance in differentiating malignant and benign nodules.

## Introduction

Lung cancer remains the most common cancer accounting for 11.6% of all diagnosed cancer cases and causes about 1.8 million cancer deaths with the highest cancer death rate of about one in five (18.4%) among all cancer deaths in 2018 worldwide (1). With an overall 5-year survival rate of only 19.4%, patients with lung cancer have a 5-year relative survival rate of 57.4% for localized stage disease, but only 16% were diagnosed at the localized stage (2). Early detection and accurate diagnosis of suspicious lung nodules are the key to minimize the mortality of lung cancer. So far, a National Lung Screening Trial (NLST) showed that low-dose CT (LDCT) screening detected 13% more lung cancer and resulted in 20% decrease in lung cancer–specific 5-year death rate than radiography (3), whereas another lung cancer screening trial (Dutch-Belgian NELSON) also revealed that LDCT screening reduced over 25% in mortality (4). Based on these encouraging results, now the annual LDCT screening for lung cancer has been routinely recommended for the elderly with high risk of lung cancer worldwide.

However, here comes another problem that a large number of incidental nodules were detected during the LDCT screening for lung cancer. How to efficiently and effectively manage this vast number of detected indeterminate nodules and identify those patients with highly suspicious nodules for close follow-up or further intensive diagnostic workup poses a great challenge to clinicians. Since as early as the late 1980s, a computer-assisted diagnosis system (CADx) has emerged and has been constantly improved aiming to enhance the clinical workflow of lung nodule management (5). To date, multiple studies (6, 7), including our previous work (8), have shown the superiority of CADx in detecting nodules on CT images with higher sensitivity than that of conventional double reading by radiologists. However, its diagnostic performance in differentiating benign and malignant nodules still needs to be further investigated.

Traditionally, using hand-engineered features such as shape features, texture features, and so on, CADx has already achieved promising performance in classifying malignant and benign lung nodules (9–11). In the latest years, deep features learned using deep convolutional neural networks (CNNs) have been shown better than hand-engineered features in multiple computer vision competitions (12–14). By applying CNN extracting deep features, a handful of studies have shown that their deep learning models had unbelievably robust classification performance in differentiating malignant and benign lung nodules (15, 16). However, most of the deep learning–based CADx (DL-CADx) are trained using publicly available database, i.e., LIDC/IDRI (Lung Image Database Consortium/Image Database Resource Initiative) database, in which histopathology of each nodule (“ground-truth”) is not available and its malignancy risk is mainly stratified by experienced radiologists (15, 17, 18). Therefore, to test its generalizability and accuracy, DL-CADx with previously reported seemingly robust classification performance should be further externally validated with pathologically confirmed lung nodules with heterogeneity from different institutions.

Herein, in this study, using confirmed histopathology as gold standard, we investigated the diagnostic accuracy of a state-of-the-art commercially available DL-CADx on a sample from our institution in differentiating benign and malignant lung nodules, compared with the performance of conventional double reading by radiologists. In addition, we also investigated the added value of objective quantifications by DL-CADx to the conventional morphology-based differential diagnosis.

## Materials and Methods

### Study Population

**FIGURE 1:**
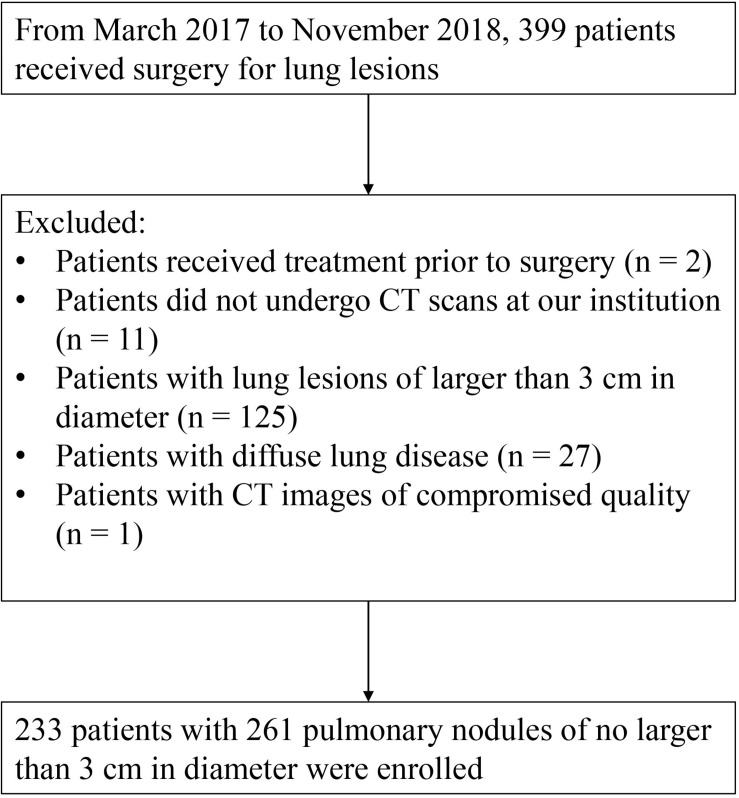
Flowchart shows the process of enrolling subjects.

This retrospective study was approved by the Institutional Review Board with the written informed consent waived from each subject. Based on the electronic medical records, from March 2017 to November 2018, 399 subjects who received surgical resection for lung lesions at our institution were enrolled. We excluded those subjects (1) who received any other cytotoxic treatment before surgery, (2) who did not take CT examination before surgery at out institution, (3) with lung lesions larger than 30 mm in diameter, (4) with significantly diffuse morphologic changes in lung parenchyma other than nodules, and (5) with quality-compromised CT image. Finally, 233 patients with 261 pathologically confirmed lung nodules were enrolled in this study (Figure 1 and Table 1).

**TABLE 1:** Patient characteristics of enrolled subjects.

**Characteristics**	**Number of patients or nodules**
Gender (male/female)	121:112
Benign group	28:16
Malignant group	93:96
Age (years)	55.87 ± 10.79
Benign group	53.0 ± 11.1
Malignant group	56.8 ± 10.6
**Size (mm)**	
Benign nodules group	13.4 ± 7.6
Malignant nodules group	16.7 ± 7.7
**Benign nodules (59)**	
Inflammatory nodule	35
Pulmonary harmatoma	4
Pulmonary nerve sheath tumor	2
Sclerosing pneumocytoma	2
Atypical alveolar hyperplasia	5
Atypical adenomatous hyperplasia	11
**Malignant nodules (202)**	
Carcinoma *in situ*	23
Minimally invasive adenocarcinoma	8
Adenocarcinoma	148
Squamous cell carcinoma	12
Small cell carcinoma	2
Mucoepidermoid carcinoma	1
Poorly differentiated carcinoma	1
Pleomorphic carcinoma of lung	1
Metastasis	6

### CT Parameters for Image Acquisition

All the CT examinations were performed either on a 64-row multi-detector CT (Optima CT660; GE Healthcare) or a 256-row multi-detector CT (Revolution; GE Healthcare) with tube voltage of 120 kVp and an automatic smart milliampere setting of tube current from 200 to 500 mA. Other parameters included matrix size = 512 × 512 pixels for both CT scanners, and collimation = 64 × 0.6 mm for the 64-row multi-detector CT and 256 × 0.6 mm for the 256-row multi-detector CT, respectively. Based on a volumetric acquisition, a bone recon type was used for image reconstruction at a slice thickness of 1.25 mm with 0.625 mm reconstruction increment. At the end of a maximal inspiration, patients received scanning from the apex of the lung to the diaphragm within a single breath-hold.

### Image Analysis

#### Double Reading by Radiologists

Blind to the results by the DL-CADx and the pathological results, two radiologists (ZL and LL with 8 years and 15 years of experience in lung CT images interpretation, respectively) independently rated the initial malignancy risk of each lung nodule using a four-scale malignancy score system in a structured report, in which 1 = unlikely to be malignant (≤25%), 2 = the likelihood of malignancy cannot be completely excluded (25–50%), 3 = highly likely to be malignant (50–75%), and 4 = considered as malignant (>75%). Then, a radiologist (DL) with more than 30 years of experience in lung imaging finalized the malignancy rating with any disagreement resolved through discussion. During evaluation, radiologists did not have access to the patients’ information, including age, gender, clinical manifestations, and laboratory test results. To mimic clinical scenarios, no specific diagnostic criteria were predefined, and the malignancy likelihood for each nodule was rated solely based on the visual assessment of the nodule morphology and manual measurement of the nodule size, CT value, and so on. When multiple nodules were detected on a single CT volume, each of nodules with size of larger than 5 mm in diameter in the axial section were evaluated separately.

Subsequently, the following information from each CT scan was recorded by conventional double reading with any disagreement resolved through discussion: (1) the location of each nodule; (2) the size of each nodule (the largest diameter and its vertical diameter of the nodule in the axial section with its largest area, and the size of part-solid nodules including the size of solid component and ground-glass component as a whole); (3) density characterization of each nodule [solid, subsolid including part-solid, and ground-glass nodules (GGNs)], followed by a list of predefined morphologic features, including (1) shape (round or oval, or irregular including triangular or polygonal); (2) margins (ill-defined or well-defined, presence of lobulation or not, presence of spiculation or not); (3) cavitation; (4) calcification; (5) air bronchogram; (6) bubble-like lucency; (7) retraction of pleura or fissure; 8) vascular convergence.

#### Evaluation by DL-CADx

A commercial DL-CADx (σ-Discover/Lung; 12 Sigma Technologies) was used to evaluate the whole set of CT images for each subject. Initially, this DL-CADx was trained on public databases, i.e., LIDC/IDRI (19) and the National Cancer Institute NLST (3). Among all the lung nodules detected for each subject, only the nodule with pathological result (the target nodule) was focused on. The first step was to check whether the target nodule on each CT was successfully detected by DL-CADx. As for nodule density subtype, the gold standard was finalized by combining both the result by DL-CADx and the result by double reading. Then, whether each target nodule successfully detected by DL-CADx was correctly subtyped into one of the three subtypes (solid, part-solid, GGN) by DL-CADx was checked, with gold standard as reference. Next, more information provided by DL-CADx was obtained, including which lobe was located, three-dimension (3D) standard diameter (the diameter of a sphere equivalent to the volume of the nodule), volume, average CT value, 3D long-axis diameter (the largest diameter in any plane of a nodule), 3D short-axis diameter (the shortest diameter vertical to the 3D long-axis diameter), and malignancy risk (ranging from 0 to 100%).

### Statistical Analysis

All statistical analysis was performed using SPSS version 24 (IBM, Armonk, NY, United States). Detection rate was defined as the ratio of the number of target nodules detected by DL-CADx to the total number of nodules of interest. Cohen’s kappa test was used to test the agreement between two raters in categorical variables, including nodule density subtype characterization between DL-CADx and the gold standard, double reading, and the gold standard, with kappa value <0, ≤0.2, ≤0.4, ≤0.6, ≤0.8, and >0.8 indicating less than chance, slight, fair, moderate, substantial, and almost perfect agreement, respectively (20). Intraclass correlation coefficient (ICC) was used to evaluate the agreement in malignancy risk rating between DL-CADx and double reading, with ICC value of <0.5, 0.5 to 0.75, 0.75 to 0.9, and >0.9 indicating poor, moderate, good, and perfect agreement, respectively (21). We arbitrarily define nodules with malignancy likelihood >50% as malignant nodules both for double reading and DL-CADx. Based on this criterion and the pathological result for each nodule, sensitivity, specificity, and accuracy were calculated for double reading and DL-CADx, separately. McNemar’s test was used to compare the diagnostic performance between DL-CADx and double reading. Chi-square test or Fisher exact test was used to compare the occurrence frequency of each morphologic change between benign group and malignant group, whereas independent sample *t*-test was used to compare the objective quantifications by DL-CADx between benign group and malignant group, with *P*-value < 0.05 indicating statistically significant difference. Logistic regression analysis was used to evaluate the combined diagnostic performance of multiple morphologic changes that have statistical significance between two groups and added value of objective quantifications by DL-CADx in differentiating benign and malignant nodules, with receiver operating characteristic (ROC) curve generated and area under the curve (AUC) calculated. Then, DeLong’s test was used to compare the performance of morphological changes and objective quantification by DL-CADx.

## Results

### Nodule Detection and Characterization

**FIGURE 2:**
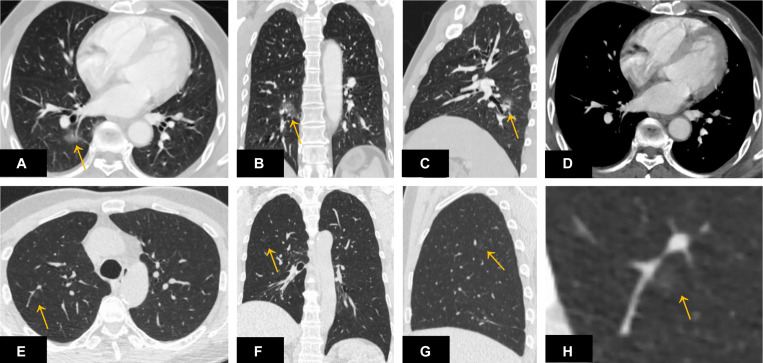
A ground-glass nodule **(A–D)** located close to the right hilum and surrounding the pulmonary vasculature was missed by DL-CADx. Another ground-glass nodule **(E–H)** smaller than 1 cm in diameter and attached to the pulmonary vasculature in the right upper lobe was missed by DL-CADx.

Among the 261 nodules, 247 nodules were successfully detected by DL-CADx with a detection rate of 94.7%. Among the 14 missed nodules, no isolated nodules were missed and 5 pleura-adjacent and 9 vessel-adjacent nodules were missed. Interestingly, regarding size, only 4 (4/13) missed nodules were smaller than 1 cm in diameter (Figure 2). As for density subtypes, 9 solid nodules, 1 part-solid nodules, and 4 GGNs were missed by DL-CADx (Figure 2). The detailed information of 14 nodules missed by DL-CADx are summarized in Table 2.

**TABLE 2:** Nodule characteristics of missed nodules by DL-CADx.

**Characteristics**	**Cases**
**Gender**	
Male	10
Female	4
**Density subtype**	
GGNs	4
Part-solid	1
Solid	9
**Localization**	
Isolated	0
Juxtapleural	5
Juxtavascular	9
**Size**	
<1 cm	4
1–3 cm	10
**Shape**	
Round or oval	5
Triangular or polygonal	9

*GGN, ground-glass nodule.*

**FIGURE 3:**
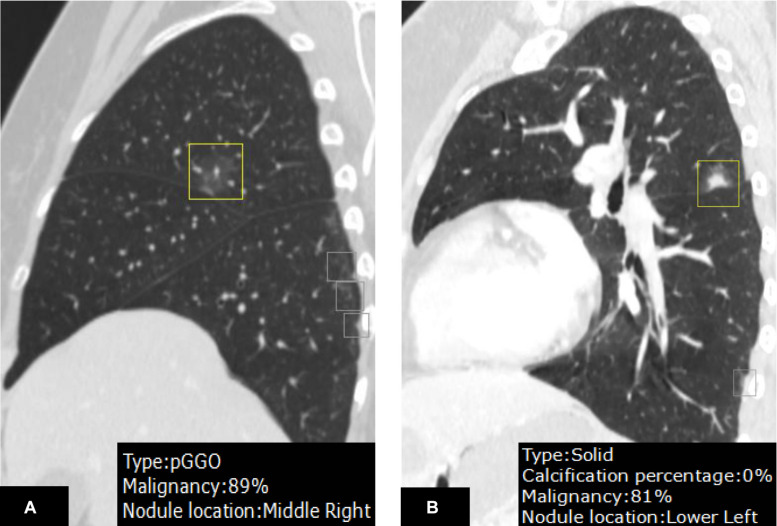
A juxtafissural nodule **(A)** located in the right upper lobe was mislabeled by DL-CADx as right middle lobe. Another nodule **(B)** attached to the fissure in the left upper lobe was mis-localized in the left lower lobe by DL-CADx.

Regarding automatic nodule localization in 5 different lobes of lung, DL-CADx almost perfectly pinpointed each nodule (214/247) in its corresponding lobe with nodule localization by double reading as gold standard (kappa value = 0.832). Particularly, it seems most challenging for DL-CADx to localize nodule close to the right middle lobe with 8 nodules in the right upper lobe and 15 nodules in the right lower lobe mistakenly labeled as nodules in the right middle lobe among all the 33 mislabeled nodules (Figure 3).

**FIGURE 4:**
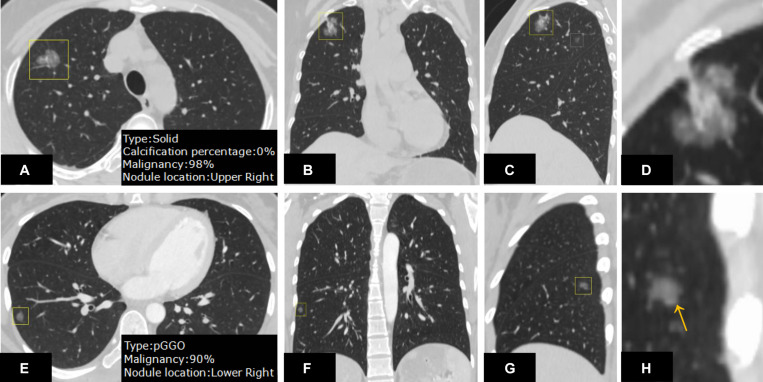
On the CT images from different views **(A–C)** and zoom-in view **(D)**, a nodule in the right upper lobe was detected. DL-CADx subtyped this nodule as a solid nodule, while double reading characterized it as a part-solid nodule. By integrating these two disagreed results, a final consensus that this is a part-solid nodule has been reached through discussion. Differently, for the nodule detected on the CT images from different views **(E–G)** and zoom-in view **(H)**, the first impression by double reading was a part-solid nodule in the left upper lobe because of seemingly a small patch of solid component (arrow). However, DL-CADx classified it as ground-glass nodule. After double checking and discussion, we misinterpreted a small vessel (arrow) inside the nodule as solid component and reached an agreement that this is a ground-glass nodule.

In nodule density subtype characterization, DL-CADx agreed substantially with double reading with subtypes of 35 of 247 nodules mismatched (kappa value = 0.747). With the final combined result as gold standard, 23 nodules (23/247) and 15 nodules (15/247) were mismatched with gold standard for DL-CADx (kappa value = 0.838) and double reading (kappa value = 0.895), respectively. Particularly, among these 23 nodules mis-subtyped by DL-CADx, 17 true part-solid nodules were subtyped as 10 solid nodules and 7 GGNs, 5 true GGNs as part-solid nodules, and only 1 true solid nodule as part-solid nodule. For 15 nodules mis-subtyped by double reading, 11 true part-solid nodules were mis-subtyped as 7 solid nodules and 4 GGNs, 3 true GGNs as part-solid nodules, and only 1 true solid nodule as part-solid nodule (Figure 4).

### Malignancy Risk Prediction

**FIGURE 5:**
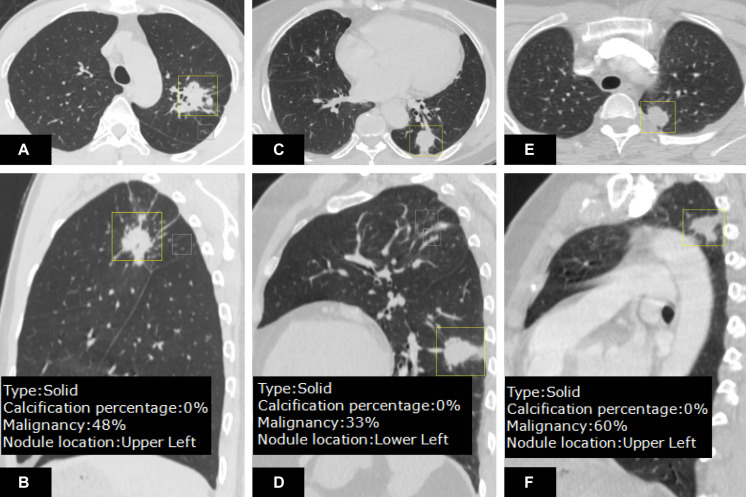
On the CT images **(A,B)**, an irregular nodule with malignancy <50% rated by DL-CADx was diagnosed as malignant by double reading (malignant likelihood: 75–100%), which turns out to be granulomatous nodule in pathology. For the nodule detected on the CT images **(C,D)**, DL-CADx considered it more likely to be a benign nodule, but double reading classified it as a malignant nodule (malignant likelihood: 75–100%), which was confirmed as adenocarcinoma. For the pathologically confirmed granuloma on the images **(E,F)**, 60% of malignancy risk was rated by DL-CADx, but double reading considered it more likely to be benign (malignant likelihood: 25–50%).

In malignancy rating, DL-CADx was in moderate agreement with double reading (ICC = 0.555, 95% CI 0.424 to 0.655). Taking malignancy likelihood of >50% as cut-off value for determining malignancy, DL-CADx misdiagnosed 40 true malignant nodules as benign nodules and 30 true benign nodules as malignant nodules with sensitivity, specificity, and accuracy of 79.2, 45.5, and 71.7%, respectively (Figure 5). In contrast, double readings achieved better performance with 16 true malignant nodules misdiagnosed as benign nodules and 26 true benign nodules as malignant nodules with sensitivity, specificity, and accuracy of 91.7, 52.7, and 83.0%, respectively (Table 3). Statistically, double reading significantly outperformed DL-CADx (*P* = 0.012).

**TABLE 3:** Comparison in diagnostic performance between DL-CADx and double reading.

**Variables**	**DL-CADx**	**Double reading**
True positive	152	176
False positive	30	26
False negative	40	16
True negative	25	29
Sensitivity	79.2%	91.7%
Specificity	45.5%	52.7%
Accuracy	71.7%	83.0%

### Quantitative Features Obtained by DL-CADx

**FIGURE 6:**
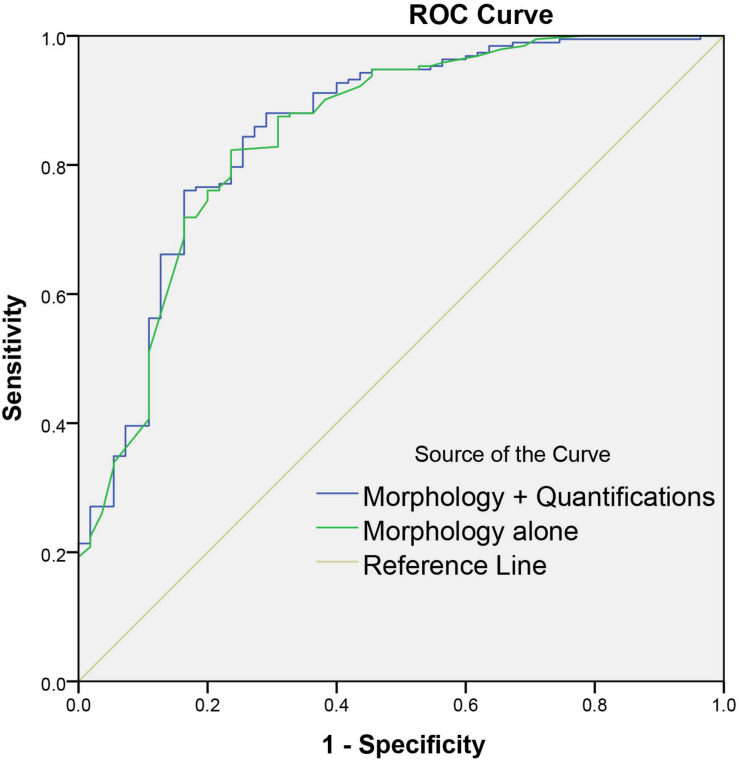
Graph shows the diagnostic performances of morphology alone and combining morphology and quantifications by DL-CADx.

Among all the morphologic features obtained by double reading, lobulation, bubble-like lucency, spiculation, pleural retraction, and vascular convergence occur significantly more frequently in malignant group than that in benign group, whereas calcification and well-defined margin are significantly more commonly detected in benign group than in malignant group (Table 4). Except for average CT value, 3D standard diameter, volume, 3D long-axis diameter, and 3D short-axis diameter were significantly different between malignant group and benign group (Table 5). In differentiating malignant and benign nodules, four quantifications by DL-CADx slightly improve the performance with AUC elevated from 0.832 (95% CI 0.762 to 0.901) for morphologic features alone to 0.840 (95% CI 0.772 to 0.909) for combining morphologic features and quantifications together (Figure 6), but without statistically significant difference (*P* = 0.412).

**TABLE 4:** Occurrence frequency difference in morphologic changes between malignant group and benign group.

	**Malignant group**	**Benign group**	***P*-value**
**Shape**			
Oval or round	61	23	0.166
Triangular or polygonal	131	32	
**Margin**			
Well-defined	182	41	0.000*
Ill-defined	10	14	
**Lobulation**			
Absence	35	31	0.000*
Presence	157	24	
**Spiculation**			
Absence	27	25	0.000*
Presence	165	30	
**Cavitation**			
Absence	187	54	0.739
Presence	5	1	
**Calcification**			
Absence	183	48	0.033*
Presence	9	7	
**Air bronchogram**			
Absence	145	43	0.683
Presence	47	12	
**Bubble-like lucency**			
Absence	132	47	0.014*
Presence	60	8	
**Pleural retraction**			
Absence	101	40	0.008*
Presence	91	15	
**Vascular convergence**			
Absence	30	39	0.000*
Presence	162	16	
**Nodule subtypes**			
GGN	51	13	0.009*
Part-solid	42	3	
Solid	99	39	

*Statistically significant (*P* < 0.05); GGN, ground-glass nodule.

**TABLE 5:** Difference in objective quantifications by DL-CADx between malignant group and benign group.

**Parameters**	**Malignant group**	**Benign group**	***P*-value**
3D standard diameter (mm)	16.2 ± 8.0	13.9 ± 7.8	0.024*
Volume (cm^3^)	4.0 ± 6.7	2.7 ± 4.8	0.011*
Average CT value (HU)	−339.2 ± 221.7	−340.9 ± 208.8	0.730
3D short-axis diameter (mm)	12.1 ± 5.7	10.4 ± 5.8	0.015*
3D long-axis diameter (mm)	19.5 ± 9.0	16.8 ± 8.8	0.039*

*Statistically significant (*P* < 0.05).

## Discussion

In this study, with histopathology of each nodule as gold standard, we have evaluated the diagnostic performance of a DL-CADx in differentiating malignant and benign lung nodules in comparison with the diagnostic performance of conventional double reading by radiologists, which indicates that although DL-CADx has shown high detection rate, its diagnostic performance in differentiating malignant and benign nodules is inferior to conventional double reading. Besides, the objective quantifications by DL-CADx added limited value to the conventional morphology-based lung nodule differentiation.

In agreement with previous studies (6, 22) and our previous work (8), DL-CADx shows high detection rate in this study. Particularly, our study indicates that it is challenging for DL-CADx to detect nodules either attached to pleura or pulmonary vasculature, consistent with previous studies (23, 24). Although automatically localizing each nodule in its corresponding lung lobe may seem an easy task for DL-CADx intuitively, DL-CADx still mislabeled a very small portion of nodules in our study. Interestingly, it seems that DL-CADx is most likely to be confused in labeling the nodules close to the middle lobe of the right lung in this study.

Nodule density subtyping is crucial in predicting its malignancy and recommending follow-up scheme, especially for part-solid nodules and GGNs, because it has been well established that most persisting subsolid nodules correspond to lung adenocarcinoma in different stages of development from atypical adenomatous hyperplasia, adenocarcinoma *in situ*, minimally invasive adenocarcinoma to invasive adenocarcinoma (25, 26). Correspondingly, 79.7% (51/64) of GGNs and as high as 93.3% (42/45) of part-solid nodules were malignant in our study. In nodule density subtyping, DL-CADx has achieved similar performance to the double reading. Interestingly, it seems most challenging for DL-CADx to accurately characterize part-solid nodules, which is consistent with the findings of our previous work (8). The “ground-truth” for the subtype of each labeled nodule for training this deep learning model is determined by expert subjectively, which is subject to inter- and intra-observer variability and let alone the highly heterogeneous data when training a deep learning model, which might explain the slight inferiority of DL-CADx in subtyping nodules than double reading. In the future, more objective criteria for nodule subtyping should be established so as to obtain “ground-truth” with higher consistency to further strengthen the deep learning model.

In disagreement with some studies which showed that DL-CADx outperformed radiologist reading (15, 16), our studies indicated that DL-CADx was still inferior to conventional double reading in differentiating malignant and benign nodules. Possible causes for the inconsistent diagnostic performance are the different deep-learning models and different training data, specifically the heterogeneous CT images acquired with different scanning parameters on different vendors. Intriguingly, DL-CADx and double reading have one thing in common that they both over-diagnosed around half of benign nodules to malignant nodules, which resulted in a relatively low specificity. For radiologists, they probably would intentionally or unconsciously raise up the malignancy level for biopsy or further diagnostic workup when a consensus cannot be reached. After all, missing a lung cancer is much more detrimental than over-diagnosing a benign nodule to malignant nodule. The DL-CADx was partly trained on the annotated lung nodules from the open public database, such as LIDC-IDRI, in which malignancy of each lung nodule was rated by experienced radiologists without pathological confirmation (15, 17, 18). The DL-CADx might have learned the tendency of over-diagnosing benign nodules to malignant nodules from radiologists. It is anticipated that with more pathologically confirmed data accumulated, a more robust deep-learning model will be trained and built. Furthermore, to train a robust DL-CADx with satisfactory generalizability, more heterogeneous datasets from different sources with confirmed pathology as “ground-truth” should be obtained in the upstream of model-building.

Although our results only showed limited additional value of the objective quantified “snapshot” of the nodule by DL-CADx to the differentiation of malignant and benign nodules, these useful objective information can be extremely useful to detect the subtle changes of nodules over time longitudinally, such as the increase of the nodule in size, change in shape and average attenuation, and newly occurred or increased solid component in a GGN or part-solid nodule. Morphology information remains dominant and reliable in differentiating malignant and benign nodules. How to effectively incorporate the general morphology information into the DL-CADx might provide another way to improve the classification performance.

## Limitations

In this study, more subjects with pathologically confirmed benign lung nodules are needed to balance the comparison. Due to possible selection bias, the accuracy obtained by DL-CADx and double reading may not represent their real accuracy. However, in this study, we only focused on how differently DL-CADx and double reading performed on the same samples. Also, to build a more robust deep learning–based CADx, the “ground-truth” of each lung nodule should be pathologically confirmed, instead of annotated by some experienced radiologists in most of the currently available deep learning–based CADx. In addition, the three different density subtypes of nodules were defined using visual assessment with no objective criteria, which might be subject to inter-observer and intra-observer variability, especially when evaluating indeterminate nodules smaller than 1 cm in diameter. Objective criteria should be defined for better characterization, so that deep-learning model could be trained on data with more accurate “ground-truth.” Besides, in this study, only one DL-CADx model was compared with conventional double reading. In the future, more studies should be done to compare the performance between more DL-CADx models and radiologists with different experience level in different clinical scenarios.

## Conclusion

Deep learning–based computer-assisted diagnosis system we used in this study presented high detection rate, great performance in subtyping lung nodules, and promising accuracy in differentiating malignant and benign nodules. However, external validation shows that its diagnostic performance is significantly inferior to conventional morphology-based double reading by radiologists. Therefore, there is still large room for improvement for the DL-CADx before it could be used to support clinical decisions in the workflow of lung nodule management. Besides, the role of objective quantification by DL-CADx in differentiating between malignant and benign nodules should be further investigated.

## Data Availability Statement

All datasets presented in this study are included in the article/supplementary material.

## Ethics Statement

The studies involving human participants were reviewed and approved by the Institutional Review Board, Department of Radiology, National Cancer Center, National Clinical Research Center for Cancer, Cancer Hospital & Shenzhen Hospital, Chinese Academy of Medical Sciences, Peking Union Medical College. The Ethics Committee waived the requirement of written informed consent for participation.

## Author Contributions

DL and ZL contributed to project idea and supervision, article revision, and integrity of this article. ZL and LL implemented the whole study, analyzed the data, and drafted the article. TL collected the raw data. DL and XW provided technical support. All authors had reviewed this article critically and approved its final submission and publication.

## Conflict of Interest

The authors declare that the research was conducted in the absence of any commercial or financial relationships that could be construed as a potential conflict of interest.

## Abbreviations

3D: three-dimensional
AUC: area under the curve
CADx: computer-assisted diagnosis system
CI: confidence interval
CNN: convolutional neural network
DL-CADx: deep learning–based computer-assisted diagnosis system
GGN: ground-glass nodule
ICC: intraclass correlation coefficient
LDCT: low-dose CT
LIDC/IDRI: lung image database consortium/image database resource initiative
NLST: National Lung Screening Trial
ROC: receiver operating characteristic.
